# Multiplexed single-molecule force spectroscopy for dissecting biophysical regulation of membrane receptors functions on live cells

**DOI:** 10.52601/bpr.2021.210022

**Published:** 2021-10-31

**Authors:** Chenyi An, Wei Chen

**Affiliations:** 1 Department of Cell Biology and Department of Cardiology of the Second Affiliated Hospital, Zhejiang University School of Medicine, Zhejiang University, Hangzhou 310058, China; 2 Key Laboratory for Biomedical Engineering of Ministry of Education and State Key Laboratory for Modern Optical Instrumentation, Zhejiang University, Hangzhou 310058, China; 3 Collaborative Innovation Center for Diagnosis and Treatment of Infectious Diseases, Zhejiang University, Hangzhou 310058, China

**Keywords:** Multiplexed SMFS techniques, Membrane receptor, Biophysical regulation, Live cell

## Abstract

Complex physical cues including two-dimensional membrane environment, dynamic mechanical force, and bioelectric activity inevitably affect membrane receptor functions. Multiplexed single-molecule force spectroscopy (SMFS) techniques with the capability of live-cell measurements are essential to systemically dissect receptor’s functions under complex biophysical regulation. In this review, we summarize recent progress of live-cell based SMFS techniques and specifically focus on the progress of SMFS on the biomembrane force probe with enhanced mechanical stability and multiplexed capability of fluorescence imaging. We further suggest the necessity of developing multiplexed SMFS techniques with simultaneous bioelectric regulation capability to investigate membrane potential regulated membrane receptor functions. These state-of-art multiplexed SMFS techniques will dissect membrane receptors functions in a systematic biophysical angle, resolving the biochemical, biomechanical and bioelectrical regulatory mechanisms in physiologically relevant conditions.

## INTRODUCTION

Complex physical cues including two-dimensional membrane environment, dynamic mechanical force, and bioelectric activity inevitably regulate membrane receptor functions. Accurately dissecting their regulatory mechanism is essential to comprehensively understand the working mechanisms of these protein machineries on the membrane. In this regard, single-molecule force spectroscopy (SMFS) techniques arm us powerful tools to characterize membrane receptor’s dynamic functions.

**Figure 1:**
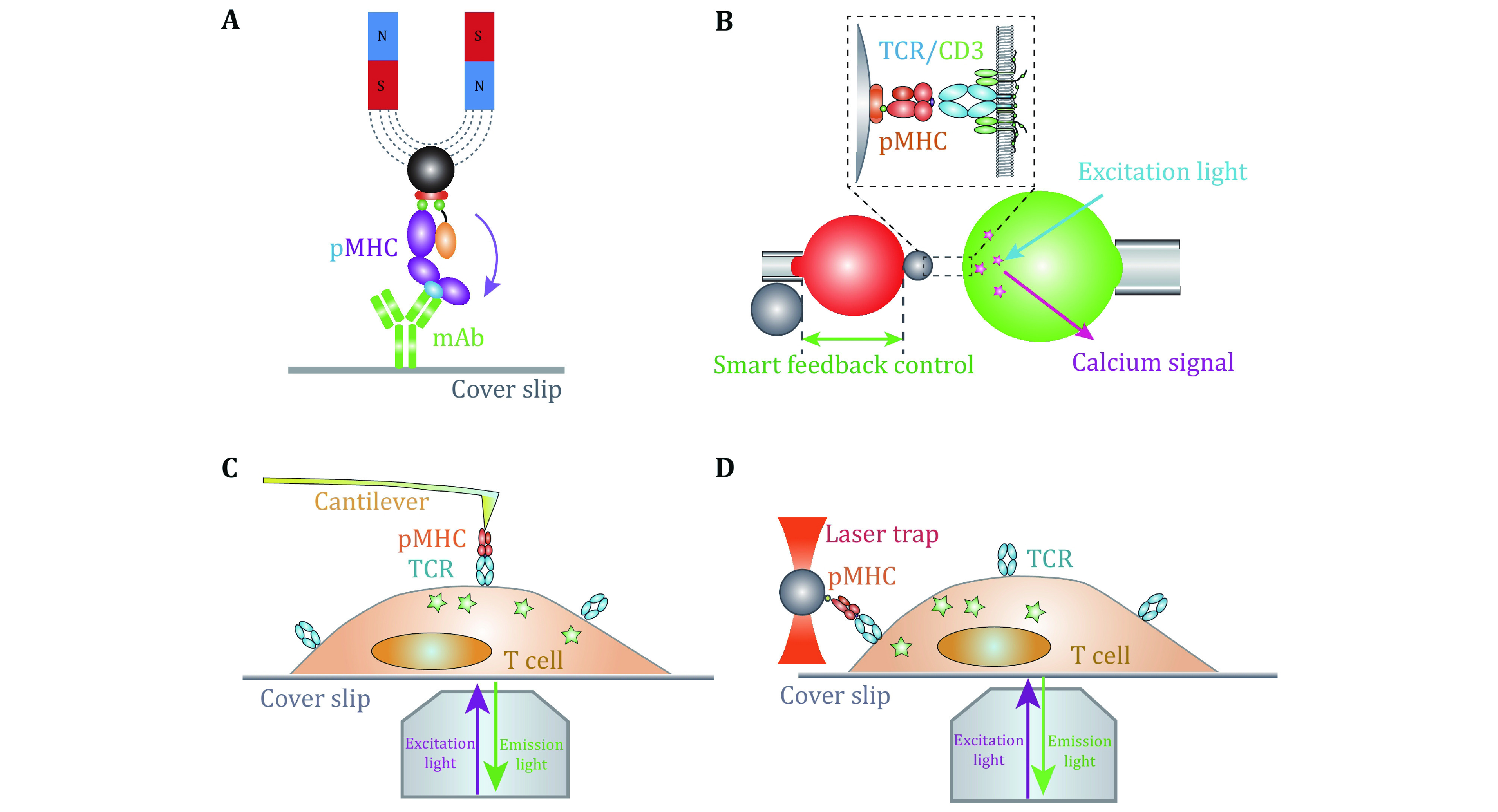
Example applications of single molecule force spectroscopy techniques in dissecting membrane receptors dynamics. **A** Magnetic tweezers dissecting the conformation changes under stretching forces (Wu *et al*. 2019). **B**–**D** Multiplexed SMFS techniques investigating the dynamic functions of *in-situ* TCR/pMHC interactions with fluorescent spectroscopy combined with BFP (**B**) (Liu *et al*. 2014), AFM (**C**) (Hu and Butte 2016) and OT (**D**) (Feng *et al*. 2017)

With the multidisciplinary supports from biophysics and bioengineering fields, it has achieved tremendous progress in the SMFS development and application in biology. SMFS techniques mainly include four platforms, atomic force microscopy (AFM), magnetic tweezers (MT), optical tweezers (OT), and biomembrane force probe (BFP) (Fig. 1). They have revolutionized biological research to reveal the recognition and activation mechanisms of membrane receptors from biophysical standpoint (Chen *et al*. 2010; Chen and Zhu 2013; Kong *et al*. 2013; Liu *et al*. 2014, 2015b; Neuman and Nagy 2008; Wu *et al*. 2019). In contrast to traditional biochemical ensemble assays, SMFS techniques integrate the advantages of “single-molecule” and “force manipulation”, capable of revealing intermediate conformational or energetic states and binding kinetics of receptor–ligand interactions at the single-molecule level and dissecting receptors’ functions under physiological-relevant forces (Chen *et al*. 2010; Ju *et al*. 2016; Liu *et al*. 2014, 2015a; Zhu *et al*. 2019). The basic principles, capabilities, and limitations of the four SMFS techniques have been reviewed in detail in (Neuman and Nagy 2008; Liu *et al*. 2015b). Among the four SMFS techniques, BFP has more appropriate spring constant (0.1–3 pN/nm) for receptor–ligand interactions than AFM (10–10^5^ pN/nm), and more convenient in incorporating live cells without immobilizing cells on substrate. However, the clamping force drifting and the relatively lower degree of automation are still bottlenecks of BFP. In this review, we mainly focus on recent progress of live-cell based SMFS techniques, especially BFP, and their applications in investigating membrane receptor’s dynamics and functions.

Plasma membrane provides an essential biophysical and biochemical platform to regulate membrane receptor functions. Traditional solution-based biophysical assays (*e*.*g*., surface plasmon resonance, SPR (Malmqvist 1993)) and purified protein based SMFS techniques are no longer appropriate to dissect membrane receptor dynamics for the following reasons. First of all, unlike the three-dimensional free diffusion and rotation of proteins in solution, two-dimensional plasma membrane exerts spatial constraint on membrane receptors, thereby limiting receptor’s conformation and spatial location and altering ligand binding kinetics (Huang *et al*. 2010; Hu *et al*. 2019). Secondly, the interactions between membrane receptors and plasma membrane (*e*.*g*., phospholipids, cholesterol) potentially regulate membrane receptor–ligand recognition (Liu *et al*. 2015a; Ma *et al*. 2017; Shi *et al*. 2013; Swamy *et al*. 2016; Zhang *et al*. 2021; Zimmerman *et al*. 2016). Thirdly, associated complex molecules in cis and downstream signaling cascades can also regulate membrane receptors’ conformations and ligand binding (Chen *et al*. 2010; Hong *et al*. 2018; Springer and Dustin 2012). Moreover, biomechanical force from traction forces during cell migration, membrane tension, and cytoskeleton contraction can also regulate membrane receptor’s conformation, ligand binding, and signaling transduction (Bashour *et al*. 2014; Chen *et al*. 2012; Hu and Butte 2016; Liu *et al*. 2014; Wang and Ha 2013; Rossy *et al*. 2018; Wu *et al*. 2019). Thus, live-cell based SMFS techniques with multiplexed biophysical manipulation and observation (*e*.*g*., mechanical force, fluorescence signal, and bioelectric activity) are required to comprehensively investigate membrane receptor dynamics and functions.

On the basis of live-cell based SMFS techniques, the development of multiplexed SMFS techniques have enabled direct investigations on mechanisms by which membrane receptor triggers cross-membrane signaling (Feng *et al*. 2017; Hu and Butte 2016; Ju *et al*. 2016; Liu *et al*. 2014) (Fig. 1B–1D). Benefiting from its compatibility of live cells and an appropriate force range (1–100 pN) for single-molecule bonds, BFP has advantages in concurrently investigating the ligand recognition and transmembrane signaling at the single-molecule level (Ju *et al*. 2016; Liu *et al*. 2014). In the following sections, we mainly focus on BFP-based SMFS techniques and summarize recent technical advances, and suggest the future development of multiplexed SMFS techniques to reveal unprecedent regulatory mechanisms of membrane receptors.

## MECHANICALLY ULTRA-STABLE BFP ENABLES ULTRA-SLOW FORCE-DEPENDENT DISSOCIATION KINETICS MEASUREMENTS ON LIVE CELLS

BFP based lived-cell SMFS has demonstrated its power to reveal the mechano-chemistry of weak-transient membrane receptor–ligand interactions, but due to mechanical drifting, characterizing strong receptor–ligand dissociation kinetics (*e*.*g*., therapeutic PD-1 antibody dissociating from membrane PD-1) under force on live cells is technically challenging (Chen *et al*. 2008, 2010), thereby inaccurately estimating ligand binding strength and functions. To overcome this, we recently developed mechanically ultra-stable BFP technique (An *et al*. 2020) (Fig. 1B). The improvements in clamping force stability and accuracy enable BFP to benchmark the ultra-slow dissociation kinetics of strong interactions between three clinical-approved PD-1 antibodies and PD-1 on live cells at the single-molecule level, revealing the better correlation of the clinical responses of the immunotherapeutic antibodies with their force-dependent dissociation kinetics than commonly accepted solution-based affinity characterized by SPR (An *et al*. 2020).

**Figure 2:**
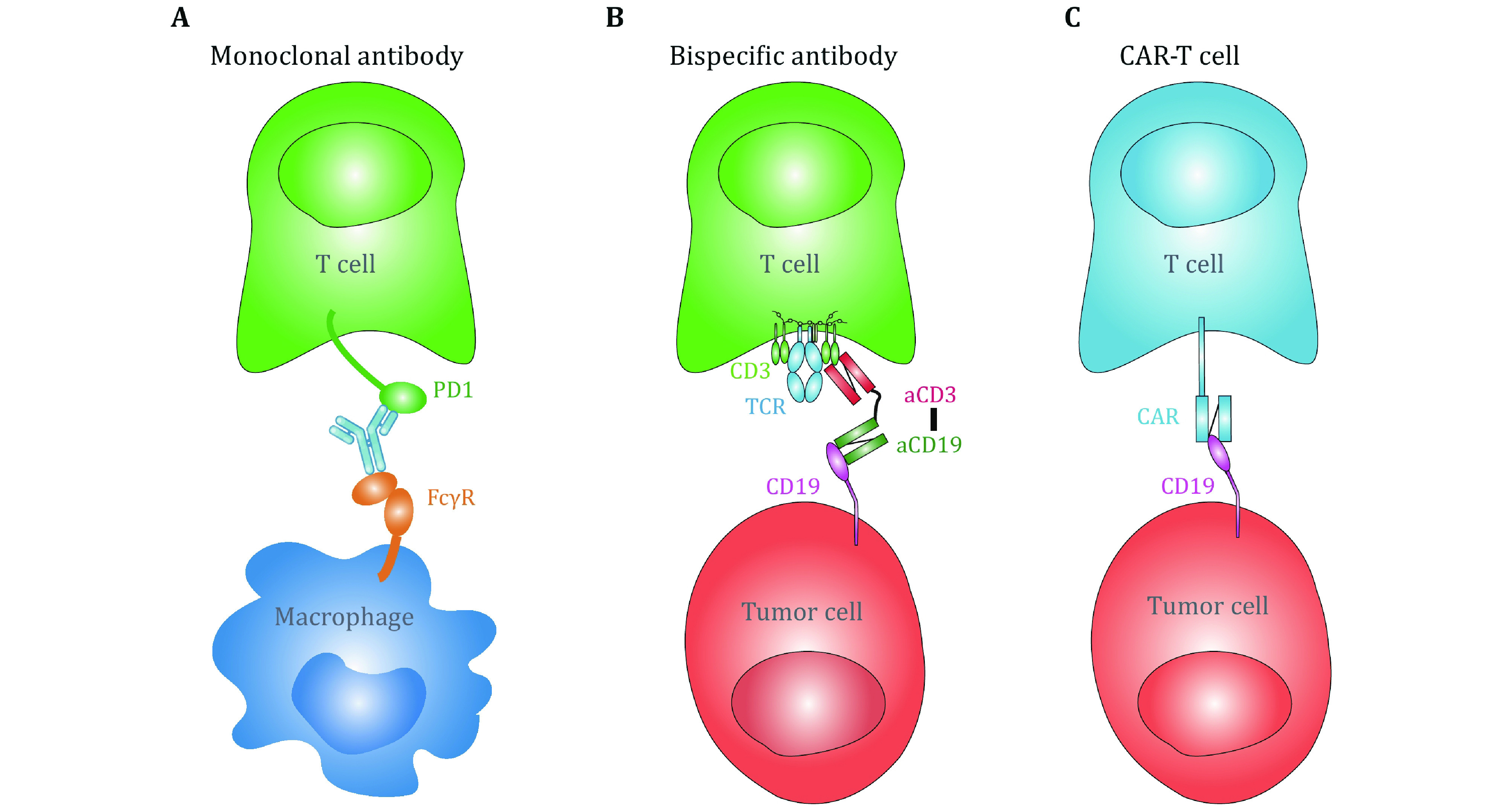
Schematics for antibody-based immunotherapies potentially sustaining biomechanical regulations. **A** PD-1 monoclonal antibody captured by FcγRs on macrophage blocks PD-1 on T cell. **B** CD3/CD19 bispecific antibody physically connect T cell with tumor cells expressing CD19. C CD19 CAR-T cell engages CD19 molecules on tumor cell

**Figure 3:**
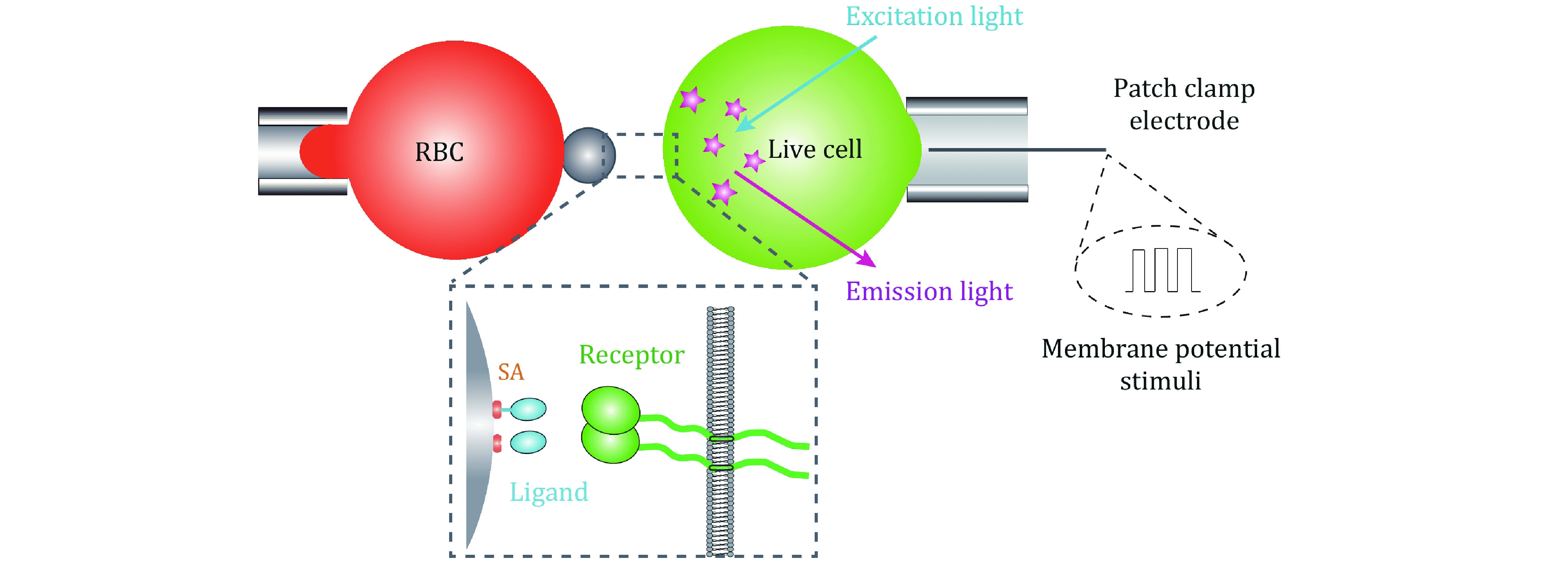
Perspective for multiplexed single molecule force spectroscopy incorporating patch clamp and fluorescent imaging techniques

With this technical advance, live-cell based SMFS measurements may potentially revolutionize immunotherapies. For example, antibody-based immunotherapies, including monoclonal antibodies (mAbs) blocking the ligand binding of immune checkpoint receptors, bispecific antibodies, and chimeric antigen receptor (CAR) T cells, have efficiently boosted cancer therapy (June *et al*. 2018; Labrijn *et al*. 2019; Pardoll 2012). One of the bottlenecks impeding accurate prediction of the clinical efficacies of these antibody-based immunotherapies is the inconsistence between the binding kinetics derived from purified proteins based assays (*e*.*g*., SPR) and functional/clinical outcomes (An *et al*. 2020; Drent *et al*. 2019; Ghorashian *et al*. 2019). For example, CAT CAR, whose solution-based binding affinity to CD19 is >40-fold lower than that of FMC63 CAR in SPR measurements, has significantly stronger cytotoxicity to CD19-expressing cell line and better clinical outcome (Ghorashian *et al*. 2019), potentially indicating the requirement of live cell in the kinetic measurements. More importantly, in the following circumstances: (1) potential capture of monoclonal antibodies by Fcγ receptors expressed on myeloid cells (An *et al*. 2020; Arlauckas *et al*. 2017) (Fig. 2A), (2) bispecific antibodies physically connect two receptors on opposing cell membranes (de Gast *et al*. 1995; Brinkmann and Kontermann 2021) (Fig. 2B), and (3) CAR-T recognition of antigenic molecules on target cells (Li et al. 2020; Porter *et al*. 2016) (Fig. 2C), biomechanical force generated from membrane tension, from cytoskeleton and from cell migration inevitably impose regulatory effects on antibody–antigen binding kinetics. In this consideration, the mechanically ultra-stable BFP technique has excellent potentials in immunotherapeutic applications, such as characterizing the force-regulated antibody–antigen binding kinetics on live cells to provide physiological-relevant biophysical parameters and optimize therapeutic efficacies (An *et al*. 2020).

## MULTIPLEXED SMFS TECHNIQUES FOR INVESTIGATING MECHANO-CHEMISTRY OF MEMBRANE RECEPTORS

Live-cell based SMFS techniques provide the capability to investigate the triggering and transmembrane signaling of membrane receptors. Although SMFS techniques are efficient in revealing ligand recognition process and/or the activation of membrane receptors *per se*, they are hard to directly observe how membrane receptors react to ligand engagement and transduce the biomechanical stimulations into biochemical signaling cascades inside cells. In this regard, Zhu and colleagues integrated fluorescent imaging with BFP to simultaneously monitor receptor–ligand binding and induced triggering signals (*e*.*g*., Ca^2+^), enabling to uncover the mechanotransduction of membrane receptors resulted from ligand binding under dynamic mechanical force application (Liu *et al*. 2014). With the fluorescent spectroscopy integrated BFP, Zhu *et* *al*. successfully revealed the digital response of force-regulated receptor–ligand binding induced receptor triggering on live T cells (Liu *et al*. 2014) (Fig. 1B) and platelets (Ju *et al*. 2016). Nevertheless, multiplexed BFP with higher imaging resolution (*e*.*g*., confocal, TIRF) are still demanded. Besides BFP, AFM (He *et al*. 2012; Hu and Butte 2016), OT (Feng *et al*. 2017; Kim *et al*. 2009; Brazin *et al*. 2018) and MT (del Rio *et al*. 2009; Guo *et al*. 2015) have also been integrated with fluorescent spectroscopy successfully. Multiplexed AFM revealed the requirement of biomechanical force in TCR triggered T cell activation (Hu and Butte 2016) (Fig. 1C). Lang *et al*. determined the direction-dependent biomechanical thresholds for TCR triggering with multiplexed OT (Feng *et al*. 2017) (Fig. 1D).

## FUTURE TRENDS FOR SMFS TECHNIQUES

Bioelectrical signals are also intrinsic biophysical modulators mediating cellular functions not only in excitable cells but also those non-excitable cells (Yang and Brackenbury 2013). Besides the well-known electrophysiological phenomena in neurobiology, bioelectrical signals have been found to execute crucial modulations in both tumorigenesis and regeneration processes (Chang and Minc 2014; Levin 2021). Although current molecular investigations on bioelectrical mechanisms are mainly confined to ion channels and cytoplasmic cascades (Chang and Minc 2014; Levin 2021), the physiological and pathological alterations of bioelectrical microenvironments potentially regulate the recognition and activation of membrane receptors through mediating the dynamics of receptors, receptor-binding proteins, phospholipids, and ionic concentrations surrounding the plasma membrane (Shi *et al*. 2013; Zhou *et al*. 2015). Revealing the interplay of biomechanical, bioelectrical, and biochemical regulatory mechanisms of membrane receptors would further uncover the functional mysteries of membrane receptors and potentially provide new strategies for biomedical applications to conquer cancer and other diseases.

In order to systematically investigate the complex biophysical regulatory mechanisms of membrane receptors, the multiplexed SMFS techniques require the incorporation of electrophysiological spectroscopy. Upon integrating SMFS techniques with patch clamp, which is efficient in controlling plasma membrane potential (*V*_m_) over the entire cell membrane with its whole-cell mode (Hamill *et al*. 1981), the biomechanical–bioelectrical coupling regulatory mechanism on membrane receptor–ligand biochemistry would be comprehensively dissected. In addition, further integrating fluorescent imaging technique would uncover membrane receptors’ transmembrane signal transduction modulated by the intersected biomechanical, bioelectrical, and biochemical cues (Fig. 3). In this way, the development of multiplexed SMFS techniques would potentially uncover membrane receptors functions in a systematic biophysical angle and revolutionize relevant biomedical applications.

Besides, the low efficiencies of SMFS techniques are still bottlenecks hindering their applications in dissecting membrane receptors functions. SMFS experiments require specialized skills and are labor-consuming due to the low-adhesion probability requirement for single-molecule bond (Johnson and Thomas 2018). Although automated AFM and high-throughput MT have partially resolved the time-consuming issues of SMFS techniques (De Vlaminck *et al*. 2011; Ribeck and Saleh 2008; Struckmeier *et al*. 2008), improving the maneuverability and throughput of SMFS experiments, especially BFP-based SMFS, is urgently demanded. In this regard, high-throughput tools, such as microfluidic devices, are potential choices to integrate with SMFS techniques in order to enhance their automation and throughput, which would benefit biological discoveries to a larger extent.

## Conflict of interest

Chenyi An and Wei Chen declare that they have no conflict of interest.
